# Role of protease activated receptor-2 in lymph node metastasis of uterine cervical cancers

**DOI:** 10.1186/1471-2407-8-301

**Published:** 2008-10-20

**Authors:** Israt Jahan, Jiro Fujimoto, Syed Mahfuzul Alam, Eriko Sato, Teruhiko Tamaya

**Affiliations:** 1 Department of Obstetrics and Gynecology, Gifu University School of Medicine, 1-1 Yanagido, Gifu City 501-1194, Japan

## Abstract

**Background:**

Protease activated receptor-2 (PAR-2) has been implicated in cellular proliferation, invasion and metastasis in various tumors. Lymph node metastasis is an important patient prognostic factor for uterine cervical cancers. This prompted us to study the role of PAR-2 in lymph node metastasis of uterine cervical cancers.

**Methods:**

Thirty patients underwent surgery for uterine cervical cancers. PAR-2 histoscores and mRNA levels were determined by immunohistochemistry and real-time reverse transcription-polymerase chain reaction, respectively. Patient prognosis was analyzed with a 48-month survival rate.

**Results:**

PAR-2 histoscores and mRNA levels significantly (*P *< 0.05) increased in 12 of 30 metastatic lymph node lesions from the corresponding primary tumor. The 48-month survival rate of the 12 patients with increased PAR-2 levels in metastatic lymph nodes was 42%, while the rate of the other 18 patients with no change in PAR-2 levels was 82%, regardless of histopathological type.

**Conclusion:**

PAR-2 might work on lymph node metastasis of uterine cervical cancers, and is considered to be a novel prognostic indicator for uterine cervical cancers.

## Background

The conventional role of serine proteases in tumor biology has changed with the recent discovery of a family of protease activated receptors (PARs). Beside contribution to cancer progression, by the degradation of extracellular matrix protein, it is now clear that some proteases serve as signal molecules controlling cell functions through this new class of receptors; which belongs to the superfamily of the serpentine G protein-coupled receptors. These receptors are activated by serine proteases that cleave within the extracellular amino terminus to expose a tethered ligand domain [1]. The new ligand binds to the core of the receptor and initiates signal transduction that results in mitogen-activating protein kinase activation [2-4], cytosolic calcium mobilization [5,6], and cell proliferation [7,8]. Macromolecular assembly and generation of serine proteases on cellular surfaces are largely involved in the regulation of the metastatic cascade [9]. Most importantly, it may provide a framework to define the role of distinct PARs in angiogenesis and tumor metastasis. The second member of the PAR family, PAR-2, is activated mainly by trypsin-like proteases [1]. The gene encoding human PAR-2 was isolated from a human genomic cDNA library using hybridization to a probe derived from the 3' exon of the mouse PAR-2 gene [10] and subsequently cloned from human kidney cDNA [10,11] localized to chromosome 5q^13 ^[12].

PAR-2 is widely expressed in the gastrointestinal tract, pancreas, kidney, liver, lung, vasculature, eye, prostate, ovary and uterus [11,13]. PAR-2 expression has also been observed in cancers of the lungs [14], liver [15], prostate [11,16], thyroid [15], breast [15,17], gastrium [18-20], colon [21,7], pancreas [22,23], gallbladder [24], and melanoma [16] and glioblastoma [25]. PAR-2 mRNA expression in lung cancers, increased by 16 fold in pulmonary tumor alveolar walls, compared to normal alveolar tissues [14]. In breast cancers, there is an upregulation of PAR-2 in proliferating stromal fibroblasts surrounding the carcinoma cells [15]. In gastric cancer cells, MKN-1, trypsin stimulates an integrin α5β1-dependent adhesion to fibronectin and proliferation through PAR-2 [20]. PAR-2 promotes tumor cell proliferation of the colon [7,21], gastrium [19,20], pancreas [22,23], and glioblastoma [25]. PAR-2 has been implicated in invasion and metastasis in cancers of the breast [15,17], colon [7,21], gastrium [19,20], pancreas [22,23], lungs [14], prostate [16], and melanoma [16], and glioblastoma [25].

The presence of lymph node metastasis, recognized as the most common metastatic lesion, is critical to patient prognosis in uterine cervical cancers [26-29]. Elucidation of the role of PAR-2, the manner of its expression in primary tumor and the corresponding metastatic lymph node lesions of uterine cervical cancers were studied.

## Methods

### Patients and tissues

Prior informed consent for the following studies was obtained from all patients and approval was given by the Research Committee for Human Subjects, Gifu University School of Medicine. Thirty patients ranging from 28 to 74 years of age underwent surgery for cervical cancer stage IIb (21 cases of squamous cell carcinoma, 5 cases of adenocarcinoma, and 4 cases of adenosquamouscarcinoma) at the Department of Obstetrics and Gynecology, Gifu University School of Medicine, between January 2000 and April 2004. Patient prognosis was analyzed in relation to 48-month survival rate. None of the patients had received any pre-operative therapy. All cases involved direct extension to the parametrium histopathologically, and had lymph node metastasis. Perfect lymphadenectomy was performed and all lymph nodes taken were divided into two. After surgery, the conventional radiotherapy for lymph node metastasis was given as adjuvant therapy. The chemotherapy with Mitomycin C, Vincristin and Cisplatin was performed when recurrence. The tissues of cervical cancer were obtained immediately after surgery. The tissues for RNA isolation were snap-frozen and stored at -80°C, and those for immunohistochemistry were fixed with 10% formalin and embedded in paraffin wax. The clinical staging of cervical cancers was determined by International Federation of Gynecology and Obstetrics (FIGO) classification [30].

### Immunohistochemistry

Four-μm sections of formalin-fixed paraffin-embedded tissue samples from cervical cancers were cut with a microtome and dried overnight at 37°C on a silanized-slide (Dako, Carpinteria, CA, USA). The protocol of universal Dako Labelled Streptavidin-Biotin kit (Dako, Carpinteria, CA, USA) was followed for each sample. Samples were deparaffinized in xylene at room temperature for 30 minutes, rehydrated with graded ethanol and washed in phosphate buffer saline (PBS). The samples were then placed in 10 mM citrate buffer (pH 6.0) and boiled in a microwave for 10 minutes for epitope retrieval. Endogenous peroxidase activity was quenched by incubating tissue sections in 3% H_2_O_2 _for 10 minutes. The primary antibody, goat PAR-2 (C-17, Santa Cruz Biotechnology, Santa Cruz, CA, USA) was used overnight at 4°C at a dilution of 1:100. The slides were washed and biotinylated anti-goat secondary antibody (Dako, Carpinteria, CA, USA) was applied for 30 minutes. After rinsing in PBS, streptavidin-conjugated horseradish peroxidase (Dako, Carpinteria, CA, USA) was added for 30 minutes. Slides were then washed and treated with the chromogen 3, 3'- diaminobenzidine (Dako, Carpinteria, CA, USA) for 5 minutes, then rinsed in PBS, and counterstained with Mayer's hematoxylin, dehydrated in graded ethanols, cleared in xylene and cover-slipped with a mounting medium, Entellan New (Merck, Darmstadt, Germany).

For the negative control of PAR-2 goat pre-immune animal serum (Dako, Carpinteria, CA, USA) was used instead of the primary antibody.

### Assessment of histochemical score (histoscore)

All sections of immunohistochemical staining for PAR-2 were evaluated in a semiquantitative fashion by two pathologists according to the method described by McCarty et al. [31], which considers both the intensity and the percentage of cells stained at each intensity. Intensities were classified as 0 (no staining), 1 (weak staining), 2 (distinct staining), 3 (strong staining) and 4 (very strong staining). For each stained section, a value designated histoscore was obtained by application of the following algorithm: histoscore = ∑(*i*+1) × *Pi*, where *i and Pi *represent intensity and percentage of cells that stain at each intensity, respectively, and corresponding histoscores were calculated separately.

### Preparation of standard template for real-time polymerase chain reaction (PCR)

Internal standard template for real-time PCR was produced by PCR amplification using the primers of PAR-2 gene, 511–947 in the cDNA (PAR-2-TS: 5'-CTCCTCTCTGTCATCTGGTT-3' and PAR-2-TAS: 5'-CTGATCATCAGCACATAGGC-3'). The DNA template was purified using a GeneClean II kit (Qbiogene, Irvine, CA, USA). The copy numbers of the standard template were determined to quantitate PAR-2 mRNA level in samples for real-time reverse transcription (RT)-PCR.

### Real-time RT-PCR

Total RNA was extracted with the acid guanidinium thiocyanate-phenol-chloroform method [32]. The total RNA (3 μg) was reverse transcribed using Moloney murine leukemia virus reverse transcriptase (MMLV-RT, 200 U/μl, Invitrogen, Carlsbad, CA, USA) and the following reagents: 250 mM Tris-HCl, pH 8.3, 375 mM KCl, 15 mM MgCl_2_, 0.1 M dithiothreitol, 10 mM deoxynucleotide [deoxyadenosine, deoxythymidine, deoxyguanosine and deoxycystidine] tri-phosphates (dNTPs) mixture and random hexamers (Invitrogen) at 37°C for 1 hour. The reaction mixture was heated for 5 min at 94°C to inactivate MMLV-RTase.

Real-time PCR reaction was performed with a Takara Ex Taq R-PCR kit, version 1.0 (Takara, Otsu, Japan), using a smart cycler system (Cepheid, Sunnyvale, CA, USA). The reaction solution (25 μl) contained Takara Ex Taq HS (5 units/μl), 10×R-PCR Buffer, 250 mM Mg^++ ^solution, 10 mM dNTP mixture, Sybr green I (1:1000 dilution; Cambrex, Otsu, Japan) and 20 μM of the primers of PAR-2 gene, 622–806 in the cDNA (PAR-2-S: 5'-AGAGGTATTGGGTCATCGTG-3' and PAR-2-AS: 5'-GCAGGAATGAAGATGGTCTG-3') with the transcribed total RNA from the tissue and a serially diluted standard template. The real-time PCR reactions were initially denatured by heating at 95°C for 30 seconds, followed by 40 cycles consisting of denaturation at 94°C for 10 seconds, annealing at 55°C for 5 seconds and extension at 72°C for 20 seconds. A strong linear relationship between the threshold cycle and the log concentration of the starting DNA copy number was always shown (correlation coefficient > 0.99). Quantitative analysis was performed to determine the copy number of each sample.

### Statistical analysis

PAR-2 mRNA levels were determined from three parts taken from each tumor, and each sample was analyzed in triplicate. PAR-2 histoscores and mRNA levels were calculated using Student's *t *test. The 48-month survival rate was calculated according to the Kaplan-Meier method, and analyzed by the log-rank test. Differences were considered significant when *P *was less than 0.05.

## Results

**Figure 1:**
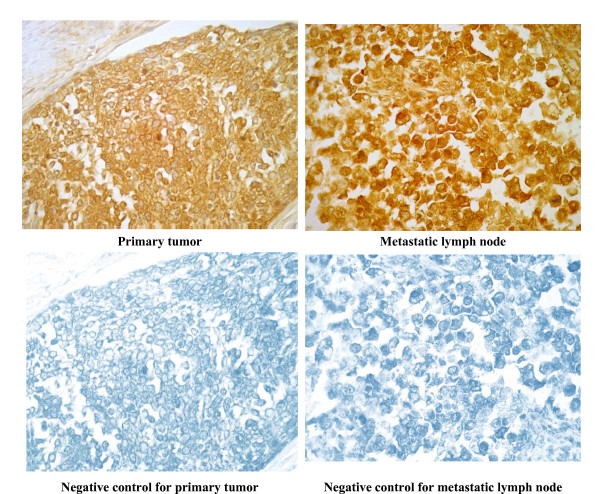
**Immunohistochemical staining for PAR-2 in primary tumor and the corresponding metastatic lymph node of uterine cervical cancers (Original magnification, ×400)**. A representative case of squamous cell carcinoma of the uterine cervix, metastatic lesion in cardinal lymph nodes. Goat anti-human PAR-2 antibody (Santa Cruz) was used at a dilution of 1: 100 as the primary antibody. Dark brown staining represents positive for PAR-2 antigen.

Immunohistochemical staining for PAR-2 on a representative case of non-keratinizing squamous cell carcinoma of the cervix, both the primary tumor and metastatic lymph node lesion are shown in Fig 1. PAR-2 was distributed in the cancer cells in all cases studied.

There was no significant difference in PAR-2 expression levels of the primary tumor of the cervix according to any clinical backgrounds: age, clinical and surgical staging, histopathology, lymph node metastasis including the number of metastatic sites and adjuvant therapy (data not shown).

**Figure 2:**
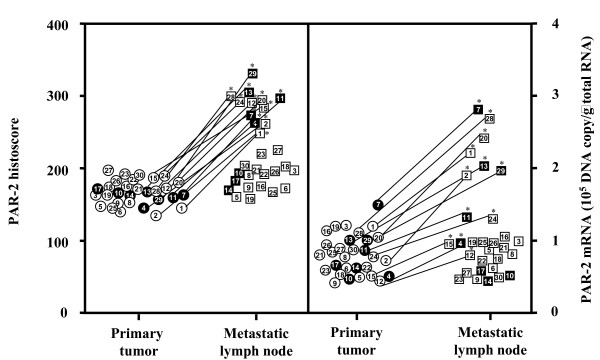
**PAR-2 histoscores and mRNA levels in primary tumor and the corresponding metastatic lymph node of uterine cervical cancers**. In the primary tumor, alive and deceased cases are numbered in open and closed circles, respectively. In the metastatic lymph node, alive and deceased cases are numbered in open and closed boxes, respectively. Each level is the mean ± SD of 9 determinations. *, *P *< 0.05 versus each primary tumor.

**Figure 3:**
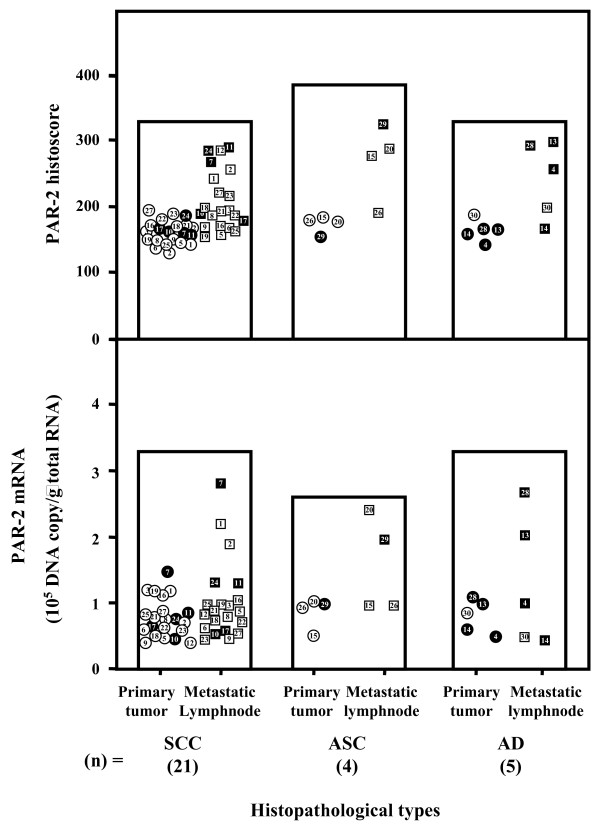
**PAR-2 histoscores and mRNA levels in primary tumor and the corresponding metastatic lymph node of uterine cervical cancers classified according to histopathological types**. SCC, squamous cell carcinoma; ASC, adenosquamous cell carcinoma; AD, adenocarcinoma. In the primary tumor, alive and deceased cases are numbered in open and closed circles, respectively. In the metastatic lymph node, alive and deceased cases are numbered in open and closed boxes, respectively. Each level is the mean ± SD of 9 determinations.

The cases with increased PAR-2 histoscores from primary tumor to the corresponding metastatic lymph node lesion were the same as those with increased PAR-2 mRNA levels. PAR-2 histoscores and mRNA levels in 12 of 30 metastatic lymph node lesions of uterine cervical cancers were significantly (*P *< 0.05 and *P *< 0.05, respectively) higher than in the corresponding primary tumors, while the scores and mRNA levels in the other 18 lesions were not significantly altered (Fig 2). There was no significant difference in PAR-2 histoscores and mRNA levels according to histopathological type (Fig 3).

**Figure 4:**
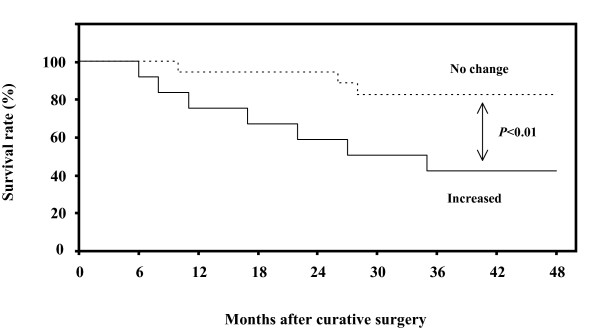
**Survival rate after surgery for uterine cervical cancers**. Patient prognosis was analyzed with a 48-month survival rate. Increased, the cases with significantly increased PAR-2 levels in metastatic lymph node lesions in comparison to the corresponding primary tumor, *n *= 12, solid line. No change, the cases with no change in PAR-2 levels in metastatic lesions in comparison to the corresponding primary tumor, n = 18, dotted line.

Patient prognosis was correlated with a 48-month survival rate. The prognosis of 12 patients with increased PAR-2 level in metastatic lesions was significantly poor (42%), while that of the other 18 patients, with unchanged PAR-2 level in metastatic lesions, was 82%. There was a significant (*P *< 0.01) difference in the 48-month survival rates between the two groups (Fig 4).

There was no significant difference concerning clinical variables such as age, histology, the number of metastatic sites, and additional therapy after surgery, except for the patient prognosis between cases of the increased PAR-2 and the unchanged PAR-2 (data not shown).

## Discussion

The present data revealed that PAR-2 significantly increased in metastatic lymph node lesions in comparison with corresponding primary tumor and correlated with poor patient prognosis in 12 out of 30 uterine cervical cancers, regardless of histopathological types. Therefore, PAR-2 might work on lymphogenous metastasis in uterine cervical cancer, and be recognized as a novel prognostic indicator for uterine cervical cancer. A plausible hypothesis might be that a cancer cell enriched PAR-2 may selectively work on lymphogenous metastasis and the expression of PAR-2 would be increased from primary tumor to the corresponding metastatic lymph node lesion. Furthermore, such a phenomenon might induce a cascade, causing PAR-2 expression to be increased in secondary metastatic lymph node lesions; resulting in poor clinical prognosis. On the other hand, in cases with no change in the PAR-2 level, PAR-2 might not directly contribute to lymph node metastasis. Although lymph node metastasis appears to be regulated by additional factors besides PAR-2, such a cascade of lymph node metastasis might be less active in these cases; resulting in comparatively better patient prognosis. This is novel evidence that PAR-2 might contribute to aggressive lymphangitic metastasis and that increase in PAR-2 from primary tumor to metastatic lymph nodes might be a prognostic indicator.

## Conclusion

PAR-2 might play an essential role as a signal to evoke and maintain the cascade of lymph node metastasis. It is further implicated for study to use PAR-2 as a tumor marker for better patient prognosis.

## Competing interests

The authors declare that they have no competing interests.

## Authors' contributions

IJ, JF and SMA contributed in experimental design. Tissue collection, RNA isolation, immunohistochemistry, RT-PCR and image preparation were performed by IJ and SMA. IJ, SMA and ES were involved in primer designing, template preparation and statistical analysis. Data interpretation and manuscript preparation and writing were done by IJ, JF and TT. The entire project was mentored and supported by JF and TT. All authors read and approved the final manuscript.

## Pre-publication history

The pre-publication history for this paper can be accessed here:


